# Impact of Risk Factors on Short and Long-Term Maternal and Neonatal Outcomes in Women With Gestational Diabetes Mellitus: A Prospective Longitudinal Cohort Study

**DOI:** 10.3389/fendo.2022.866446

**Published:** 2022-06-20

**Authors:** Antonella Corcillo, Dan Yedu Quansah, Christophe Kosinski, Katrien Benhalima, Jardena J. Puder

**Affiliations:** ^1^ Service of Endocrinology, Diabetes and Metabolism, Department of Medicine, Lausanne University Hospital, Lausanne, Switzerland; ^2^ Obstetric Service, Department Woman-Mother-Child, Lausanne University Hospital, Lausanne, Switzerland; ^3^ Department of Endocrinology, Universitair Ziekenhuis (UZ) Gasthuisberg, Katholieke Universiteit (KU) Leuven, Leuven, Belgium

**Keywords:** risk factors, gestational diabetes mellitus, maternal outcomes, neonatal outcomes, gestational diabetes, GDM

## Abstract

**Aims:**

Universal screening of gestational diabetes mellitus (GDM) in women with no risk factors (RF) for GDM remains controversial. This study identified the impact of the presence of RF on perinatal and postpartum outcomes.

**Methods:**

This prospective cohort study included 780 women with GDM. GDM RF included previous GDM, first grade family history of type 2 diabetes, high-risk ethnicity and pre-pregnancy overweight/obesity (OW/OB). Outcomes included obstetrical, neonatal and maternal metabolic parameters during pregnancy and up to 1 year postpartum.

**Results:**

Out of 780 patients, 24% had no RF for GDM. Despite this, 40% of them needed medical treatment and they had a high prevalence of glucose intolerance of 21 and 27% at 6-8 weeks and 1-year postpartum, respectively. Despite similar treatment, women with RF had more neonatal and obstetrical complications, but they had especially more frequent adverse metabolic outcomes in the short- and long-term. The most important RF for poor perinatal outcome were previous GDM and pre-pregnancy OW/OB, whereas high-risk ethnicity and pre-pregnancy OW/OB were RF for adverse postpartum metabolic outcomes. Increasing number of RF were associated with worsened perinatal and long-term postpartum outcomes except for pregnancy-induced hypertension, C-section delivery and neonatal hypoglycaemia.

**Conclusion:**

Women with no RF had a high prevalence of adverse perinatal and postpartum outcomes, while the presence of RF particularly increased the risk for postpartum adverse metabolic outcomes. This calls for a RF-based long-term follow-up of women with GDM.

## Introduction

Prevalence of gestational diabetes mellitus (GDM) is estimated to be between 3-30% (1, 2) worldwide and is associated with significant morbidity for the mother and her offspring. In Switzerland, its prevalence is around 11% (3, 4). Women with RF have a 2 to 7-fold prevalence of GDM than those without RF (RF) (2, 4). Although, there are discrepancies in European guidelines regarding the choice of RF that could serve as a base for selective GDM screening (4, 5), classical RF that are found in most guidelines are GDM in previous pregnancy, first grade family history of type 2 diabetes (FH T2DM), high-risk ethnicity and pre-pregnancy overweight or obesity (OW/OB) (5–8).

Even though universal screening is advocated by most international recommendations (9, 10), it remains controversial whether women without RF should also be screened. Many studies have compared various testing recommendations and timing of screening (11–13). Although these studies reported higher prevalence of GDM based on universal screening, its benefits on severe maternal outcomes and cost-effectiveness still remain unclear especially in limited resource settings (11–13). Benhalima et al. showed that the prevalence of GDM in women without established RF varied substantially between 50-70% when different European selective screening guidelines were applied to their cohort (5).

Several studies have shown the associations between RF for GDM and adverse perinatal and post-partum maternal and neonatal outcomes (14–18). The RF included higher oral glucose tolerance test (oGTT) values during pregnancy and in the postpartum period, HbA1c during pregnancy, paternal type 2 diabetes, multigravida, higher parity and longer interval between delivery and follow-up (14–18). However, there is a lack of long-term postpartum follow-up and no studies have investigated the impact of specific factors and of increasing number of GDM RF on perinatal and postpartum outcomes in order to stratify women according to their risk.

The aim of this study was to assess among women with GDM the prevalence of women without any classical RF and evaluate their adverse short- and long-term outcomes in a clinical context. We also sought to identify the impact of each individual RF independently on neonatal and maternal outcomes and to investigate if adverse outcomes increase with increasing number of RF. This could help to identify women who need an intensive long-term follow-up.

## Methods

### Study Design and Patient Population

This was a prospective observational cohort of women with GDM followed in the Diabetes and Pregnancy Unit at the Lausanne University Hospital in Switzerland between April 2012 and December 2017. This cohort data has been previously described elsewhere (19–25). Women were followed during pregnancy and at the early (6-8 weeks) postpartum and included a nested subcohort at late (1-year) postpartum. Of all women included, 91% had complete laboratory data at the 6-8 weeks follow-up whereas 22% had complete laboratory data at the 1-year postpartum visit. The main reason for the low numbers of patients at 1-year postpartum visit was that the implementation of the 1-year postpartum follow-up visit started only in August 2015.

### GDM Diagnosis, Treatment and Follow-Up

GDM was diagnosed according to the ‘International Association of Diabetes and Pregnancy Study Groups’ (IADPSG) and American Diabetes Association (ADA) Criteria (10). Thus, GDM was diagnosed if fasting glucose was ≥5.1 mmol/l and/or 1h glucose was ≥10.0 mmol/l and/or 2h glucose was ≥8.5 mmol/l, following a 75 g oGTT at 24-28 weeks of gestational age. The treatment of GDM was based on the current guidelines of the ADA (9) and of the Endocrine Society (7). After GDM diagnosis, women had a weekly appointment with a medical doctor, a specialized diabetes nurse and/or a dietician during which they received information about GDM, were taught how to perform a capillary blood glucose test and received more specific recommendations on lifestyle and gestational weight gain. Physical activity was encouraged and counselling by a physiotherapist and/or participation in GDM physical activity groups were proposed.

Patients were asked to perform 4 times per day self-monitoring of blood glucose according to international and local guidelines including fasting capillary glucose (FBG) in the morning and 2h (or 1h) postprandial glucose after each meal (26). Metformin and/or insulin were introduced when glucose values remained above targets between two or more times during a 1 to 2-week period (FBG > 5.3 mmol/l, 1h postprandial glucose > 8 mmol/l and 2h postprandial glucose > 7 mmol/l) despite lifestyle changes. Treatment was recommended based on glucose values (i.e. insulin in case of relatively high values), patient characteristics (i.e. BMI) and patient medical history and preference. Thus, metformin was especially used in case of patients who would refused insulin or if insulin doses were very high. Short acting insulin analogues were introduced and adapted to achieve 1h postprandial glucose ≤8 mmol/l or 2h post-prandial glucose ≤7 mmol/l and long acting insulin analogues to achieve FBG ≤5.3 mmol/l.

### Measures

#### Measures of Glycaemic Control

HbA1c during pregnancy was measured using a chemical photometric method (conjugation with boronate; Afinion^®^). The Afinion^®^ analyser has shown to have similar accuracy and precision compared to the high-performance liquid chromatography (HPLC), which is IFCC (International Federation of Clinical Chemistry and Laboratory Medicine) standardized and DCCT (Diabetes Control and Complications Trial) aligned (26). In both postpartum periods, HbA1c was measured using HPLC. HbA1c at the end of pregnancy was only performed after March 2015. Whereas FPG, 2h glucose after a 75g oGTT and HbA1c were measured in the early postpartum visit, only FPG and HbA1c were measured in the late postpartum visit. Glucose intolerance was defined as fasting glucose ≥5.6mmol/l or 2h glucose ≥7.8mmol/l or HbA1c ≥5.7% (39 mmol/mol).

#### Maternal Predictors and Outcomes Measures

The following predictors were included in this study: previous GDM history, FH T2DM, high-risk ethnicity and OW/OB before pregnancy. Maternal ethnicity was classified as low risk (Europe, North America) and high risk (Asia, Central and South America, Africa, Oceania) groups (9).

Although these predictors are not the only factors recommended by the scientific communities, they are consistent with the ADA and the National Institute for Health and Care Excellence (NICE) RF for prediabetes, type 2 diabetes and GDM (5–8). We selected them because they are measures that are reliable and easy to record in daily practice on a larger scale and are frequent enough in this age group and population to have an impact. We therefore did not include other RF such as macrosomia in a previous pregnancy [also removed in the newest ADA recommendations (9)], polycystic ovary syndrome (PCOS), history of cardiovascular disease, hypertension, hypercholesterolemia and hypertriglyceridemia. We also did not include physical inactivity in the analysis because the accuracy of these data in our cohort was not optimal. Pre-pregnancy weight was taken from participants medical charts or, if missing, was self-reported (for the 1–2 months before pregnancy) and weight was measured during pregnancy and in the postpartum period. Height was measured at the first visit at the GDM clinic, body mass index (BMI) was calculated as the ratio of weight in kilograms to the square of height in meters (kg/m2) and OW/OB was defined as BMI ≥ 25 kg/m2. Excessive gestational weight gain (GWG) up to presentation at GDM clinic was defined according to the Institute of Medicine recommendations (IOM) (27). We had valid complete data (n=780) for previous GDM history, FH T2DM, and OW/OB before pregnancy but unfortunately we had 27 out of 780 women missing data for ethnicity. Where ethnicity was not a predictor either as a single predictor or in the combined predictor scores, we included all 780 women in the analysis.

Adverse maternal outcomes including HbA1c at presentation and at the end of the pregnancy (20, 28), need for pharmacological treatment during pregnancy, C-section delivery, pre-eclampsia, pregnancy induced hypertension (PIH) and measures of glycaemic control at 6-8 weeks (defined as early postpartum) and 1 year (defined as late postpartum) were assessed. We also assessed composite outcome of maternal complications (including placenta previa and other various pregnancy related, rarer complications such as thrombopenia, chorioamnionitis). The decision for C-section delivery was taken by the patients’ obstetrician.

Adverse neonatal outcomes were preterm delivery (defined as <37 weeks of gestation), large-for- gestational age baby [LGA; as defined by Intergrowth (29)], neonatal hypoglycaemia (defined as ≤2.5 mmol/l) and a composite of adverse neonatal outcomes (including Apgar score at 5 minutes < 7 and admission to the intensive care unit).

### Statistical Analysis

All data were analysed using Stata/SE 15.0 (StataCorp LLC, TX, USA). Normally distributed continuous variables were expressed as means and standard deviation (SD). Binary outcomes were described in frequency and percentages (n, %). The results did not significantly vary with or without exclusion of nulliparous women and so nulliparous women were included in all descriptive and outcome analyses to increase external validity, except if the predictor was “GDM in previous pregnancy” (**Tables 1**–**4**). Excessive GWG up to presentation at GDM clinic was defined according to the IOM guidelines (27) and was transformed as a binary outcome. In **Table 2**, we presented raw data and differences, but we performed an additional analysis and adjusted for parity, gestational age at presentation, which were different between RF + and RF- women, and for gestational age at delivery for obstetric, neonatal and postpartum outcomes, as some of the outcomes might be influenced by this. In **Table 3**, we performed a univariate analysis with potential predictors of adverse outcomes and predictors with a p-value <0.05 were included in the multivariable logistic regression analysis model with stepwise procedure, adjusting for parity and gestational age at presentation.

**Table 1:** Descriptive characteristics of patients before pregnancy or at presentation.

	No risk factor (24%, n = 182)	At least one risk factor (76%, n = 571)	p-value
Age, years	33.4 (±5.6)	33.0 (±5.4)	0.430
Educational level			0.002
Compulsory school achieved	10 (12%)	44 (20%)	
CFC ^a^	20 (23%)	48 (22%)	
High school	6 (7%)	29 (13%)	
University	50 (58%)	84 (38%)	
Not achieved	0	16 (7%)	
Gravidity	2.0 (±1.3)	2.6 (±1.6)	< 0.001
Parity	0.5 (±0.7)	1.0 (±1.1)	< 0.001
Weight before pregnancy, kg	59.5 (±6.4)	72.5 (±16.3)	< 0.001
BMI before pregnancy, kg/m^2^	21.8 (±1.9)	27.2 (±5.6)	< 0.001
Gestational age at presentation, weeks	29.3 (±2.7)	28.4 (±3.5)	0.005
Weight at presentation, kg	82.7 (±16.3)	70.6 (±7.8)	< 0.001
Weight gain, kg	11.1 (±4.5)	10.1 (±5.8)	0.009
Excessive weight gain up to presentation at GDM clinic	129 (75%)	444 (79%)	0.175
Excess of weight gain up to presentation at GDM clinic, kg	2.9 (±4.5)	4.3 (5.6)	0.005
GDM in previous pregnancy^+^	0	61 (11%)	*n/a*
FH T2DM	0	248 (43%)	*n/a*
Ethnicity^++^ (n=753)			< 0.001
Low risk (Europe, North America, Switzerland)	182 (100%)	301 (53%)	
High risk (Africa, Central and South America, Asia, Oceania)	0	270 (47%)	
OW/OB before pregnancy	0	371 (65%)	*n/a*

Data presented as n (%) or mean (± SD). BMI, Body mass index, FH T2DM, family history with 1st degree relative with type 2 diabetes mellitus, OW/OB, overweight/obesity defined as BMI ≥ 25 kg/m2. n/a, not applicable.

For educational level, data were available for n==307.

a CFC means general and vocational education.

^+^Only patients with parity ≥1 (n= 439).

^++^Low risk ethnicity defined as Europe (n=95, 53% and n=156, 27%), North America (n=3, 1% and n=1, 1%) and Switzerland (n=84, 46% and n=144, 25%) ethnic groups for no risk factor and at least one risk factor group respectively. High risk ethnicity defined as Africa (n=125, 22%), Central and South America (n=39, 6%), Asia (n=104, 18%) and Oceania (n=2, 1%) ethnic groups.

**Table 2:** Impact of the absence or presence of any risk factors on short and long-term maternal and neonatal outcomes.

	No risk factor (n = 182)	At least one risk factor (n = 571)	OR^#^/β-coefficient(95% confidence interval)	p-value
**Maternal outcomes**				
HbA1c at presentation, %	5.3 (±0.4)	5.5 (±0.4)	0.17 (0.09 – 0.24)	< 0.001
HbA1c at presentation, mmol/mol	34.7 (±3.9)	36.5 (±4.7)	1.85 (1.06 – 2.63)	< 0.001
HbA1c at the end of pregnancy, %	5.4 (±0.4)	5.6 (±0.4)	0.13 (0.02 – 0.23)	0.018
HbA1c at the end of pregnancy, mmol/mol	36 (±3.9)	37 (±4.4)	0.36 (0.24 – 2.49)	0.018
Need for pharmacological treatment	72 (40%)	310 (54%)	1.82^#^ (1.29 – 2.55)	< 0.001
C-section delivery	59 (37%)	222 (41%)	1.22^#^ (0.85 – 1.75)	0.285
Pregnancy induced hypertension	5 (3%)	19 (3%)	1.22^#^ (0.45 – 3.31)	0.693
Pre-eclampsia	7 (4%)	7 (1%)	0.31^#^ (0.11 – 0.89)	0.031
Composite maternal complications ^a^	2 (1%)	21 (4%)	3.44^#^ (0.79 – 14.79)	0.098
**Overall glucose intolerance in the early postpartum^+^**	33 (21%)	182 (36%)	2.07^#^ (1.35 – 3.16)	0.001
**Abnormal fasting glucose at 6-8 weeks postpartum** Pre-diabetes (IFG) Diabetes	11 (7%) 11 (7%) 0	84 (17%) 76 (15%) 8 (2%)	2.68^#^ (1.39 – 5.16)	0.001
**Abnormal 2h glucose at 6-8 weeks postpartum** Pre-diabetes (IGT) Diabetes	7 (5%) 6 (4%) 1 (1%)	46 (9%) 39 (8%) 7 (1%)	2.11^#^ (0.94 – 4.78)	0.051
**Abnormal HbA1c at 6-8 weeks postpartum** Pre-diabetes Diabetes	22 (16%) 22 (16%) 0	126 (25%) 122 (24%) 4 (1%)	2.04^#^ (1.25 – 3.33)	0.003
**Overall glucose intolerance in the late postpartum^+^**	10 (27%)	68 (52%)	2.91^#^ (1.31 – 6.50)	0.006
**Abnormal fasting glucose at 1 year postpartum** Pre-diabetes (IFG) Diabetes	10 (27%) 10 (27%) 0	60 (46%) 57 (44%) 3 (2%)	2.28^#^ (1.02 – 5.09)	0.037
**Abnormal HbA1c at 1 year postpartum** Pre-diabetes Diabetes	1 (3%) 1 (3%) 0	26 (19%) 23 (17%) 3 (2%)	8.75^#^ (1.15 – 66.78)	0.004
**Neonatal outcomes**				
Preterm delivery	24 (14%)	43 (8%)	0.51^#^ (0.30 – 0.88)	0.015
LGA	16 (10%)	95 (17%)	1.95^#^ (1.11 – 3.42)	0.019
Neonatal hypoglycaemia	13 (7%)	49 (9%)	1.22^#^ (0.65 – 2.30)	0.532
Composite neonatal complications^b^	22 (16%)	60 (12%)	0.72^#^ (0.42 – 1.22)	0.236

Data presented as n (%) or mean (±SD). Odds ratio (OR) are marked with #.

Nulliparous patient were included in the analysis, as results were similar when they were excluded.

For HbA1c at presentation and at the end of pregnancy, data were available for n==298 and n=168, respectively. Early post-partum was defined as 6-8 weeks post-partum and late post-partum as 1 year post-partum. Glucose intolerance defined as fasting glucose ≥5.6mmol/l or glucose T120 ≥7.8mmol/l (only for early post-partum) or HbA1c ≥5.7% (39 mmol/mol). Preterm delivery was defined as < 37 weeks. LGA = large for gestational age. Neonatal hypoglycaemia was defined as ≤ 2.5 mmol/l.

^+^Overall glucose intolerance includes women with prediabetes and in addition 14 cases of diabetes in the early postpartum and 5 cases in the late post-partum.

a Maternal complications include various pregnancy related complications such as placenta praevia, thrombopenia,…

b Composite neonatal complications include Apgar score at 5 minutes < 7 and admission to intensive care unit (data available for n = 615).

**Table 3:** Independent impact of individual risk factors on maternal and neonatal outcomes.

	Significant risk factors	OR ^#^/β-coefficient (95%CI)	p-value
**Maternal outcomes**			
HbA1c at presentation	Previous GDM OW/OB	0.23 (0.12 – 0.35)0.17 (0.10 – 0.22)	< 0.001< 0.001
HbA1c end pregnancy, %	Previous GDM OW/OB	0.17 (-0.02 – 0.36) 0.10 (0.01 – 0.19)	0.078 0.023
Need for pharmacological treatment	FH T2DMOW/OB	1.52^#^ (1.1 – 2.1) 1.70^#^ (1.26 – 2.29)	0.009 < 0.001
C-section delivery	OW/OB	1.36^#^ (1.01 –1.83)	0.046
Pregnancy induced hypertension	OW/OB	2.48^#^ (1.00 – 6.17)	0.050
Composite maternal complications ^a^	Previous GDM	4.01^#^ (1.32 – 12.20)	0.014
Overall glucose intolerance in early postpartum^+^	Previous GDM High risk ethnicity OW/OB	2.17^#^ (1.16 – 4.04) 1.67^#^ (1.19 – 2.34) 1.67^#^ (1.19 – 2.33)	0.015 0.003 0.003
Overall glucose intolerance in late postpartum^+^	High risk ethnicity OW/OB	2.20^#^ (1.11 – 4.38) 2.45^#^ (1.29 – 4.69)	0.025 0.007
**Neonatal outcomes**			
Preterm delivery	High risk ethnicity	0.39^#^ (0.21 – 0.73)	0.004
LGA	OW/OB	1.97^#^ (1.28 – 3.03)	0.002

Stepwise multiple regression including all variables at 0.05 of significance was performed. All 4 risk factors and all outcomes were tested, but for readability only significant ones reported (p < 0.1, i.e. statistical significance was defined as a p-value < 0.1). All analyses were adjusted for parity and gestational age at presentation. Nulliparous patients were included in the analysis, as results were similar when excluded. Odds ratio (OR) are marked with #.

OW/OB= pre-pregnancy overweight or obesity. FH T2DM = family history of 1st degree with type 2 diabetes mellitus. Early post-partum was defined as 6-8 weeks post-partum (n=670) and late post-partum as 1 year post-partum (n=168). Glucose intolerance was defined as fasting glucose ≥5.6mmol/l or glucose T120 ≥7.8mmol/l (only for early post-partum) or HbA1c ≥5.7% (39 mmol/mol). Preterm delivery defined as < 37 weeks. LGA = large for gestational age.

^+^Overall glucose intolerance includes women with prediabetes and in addition 14 cases of diabetes in the early post-partum and 5 cases in the late post-partum.

a Maternal complications include various pregnancy related complications such as placenta praevia, thrombopenia,…

**Table 4:** Cumulative impact of the number of risk factors (0-4) on short and long-term maternal outcomes.

	OR^#^/β-coefficient (95%CI)	p-value
**Maternal outcomes**		
HbA1c at presentation	0.12 (0.09 – 0.15)	< 0.001
HbA1c at the end of pregnancy, %	0.08 (0.04 – 0.13)	< 0.001
Need for pharmacological treatment	1.50^#^ (1.2 – 1.7)	< 0.001
C-section delivery	1.18^#^ (1.00 – 1.40)	0.225
Pregnancy induced hypertension	1.00^#^ (0.64 – 1.55)	0.996
Pre-eclampsia	0.47^#^ (0.23 – 0.96)	0.040
Composite maternal complications ^a^	1.42^#^ (0.92 – 2.19)	0.116
Glucose intolerance in early post-partum^+^	1.39^#^ (1.16 – 1.66)	< 0.001
Glucose intolerance in late post-partum^+^	1.66^#^ (1.15 – 2.38)	0.001
**Neonatal outcomes**		
Preterm delivery	0.71^#^ (0.53 – 0.96)	0.025
LGA	1.31^#^ (1.05 – 1.64)	0.016
Neonatal hypoglycaemia	0.98^#^ (0.74 – 1.31)	0.926
Composite neonatal complications ^b^	0.94^#^ (0.73 – 1.21)	0.808

All analysis were adjusted for parity and gestational age at presentation. Nulliparous patients were included in the analysis. Odds ratio (OR) are marked with #.

For HbA1c at presentation and at the end of pregnancy, data were available for n==298 and n=168, respectively. Early post-partum was defined as 6-8 weeks post-partum and late post-partum as 1 year post-partum. Glucose intolerance defined as fasting glucose ≥5.6mmol/l or glucose T120 ≥7.8mmol/l (only for early post-partum) or HbA1c ≥5.7% (39 mmol/mol). Preterm delivery defined as < 37 weeks. LGA = large for gestational age. Neonatal hypoglycaemia defined as ≤ 2.5 mmol/l.

^+^Overall glucose intolerance includes women with prediabetes and in addition 14 cases of diabetes in the early postpartum and 5 cases in the late post-partum.

a Maternal complications include various pregnancy related complications such as placenta praevia, thrombopenia,…

b Composite neonatal complications include Apgar score at 5 minutes < 7 and admission to intensive care unit (data available for n=615).

In the logistic regression analyses, adjusted odds ratios (OR) were reported along with their respective 95% confidence intervals (CI). **Table 4** shows the results of regression analysis of the cumulative impact of the number of risk factors (0-4) on short and long-term maternal outcomes, adjusted for parity and gestational age at presentation. All statistical significances were two-sided and accepted at p<0.05 except for the multiple regression models where statistical significance was accepted at p<0.1 (in **Table 3**).

## Results

**Figure 1:**
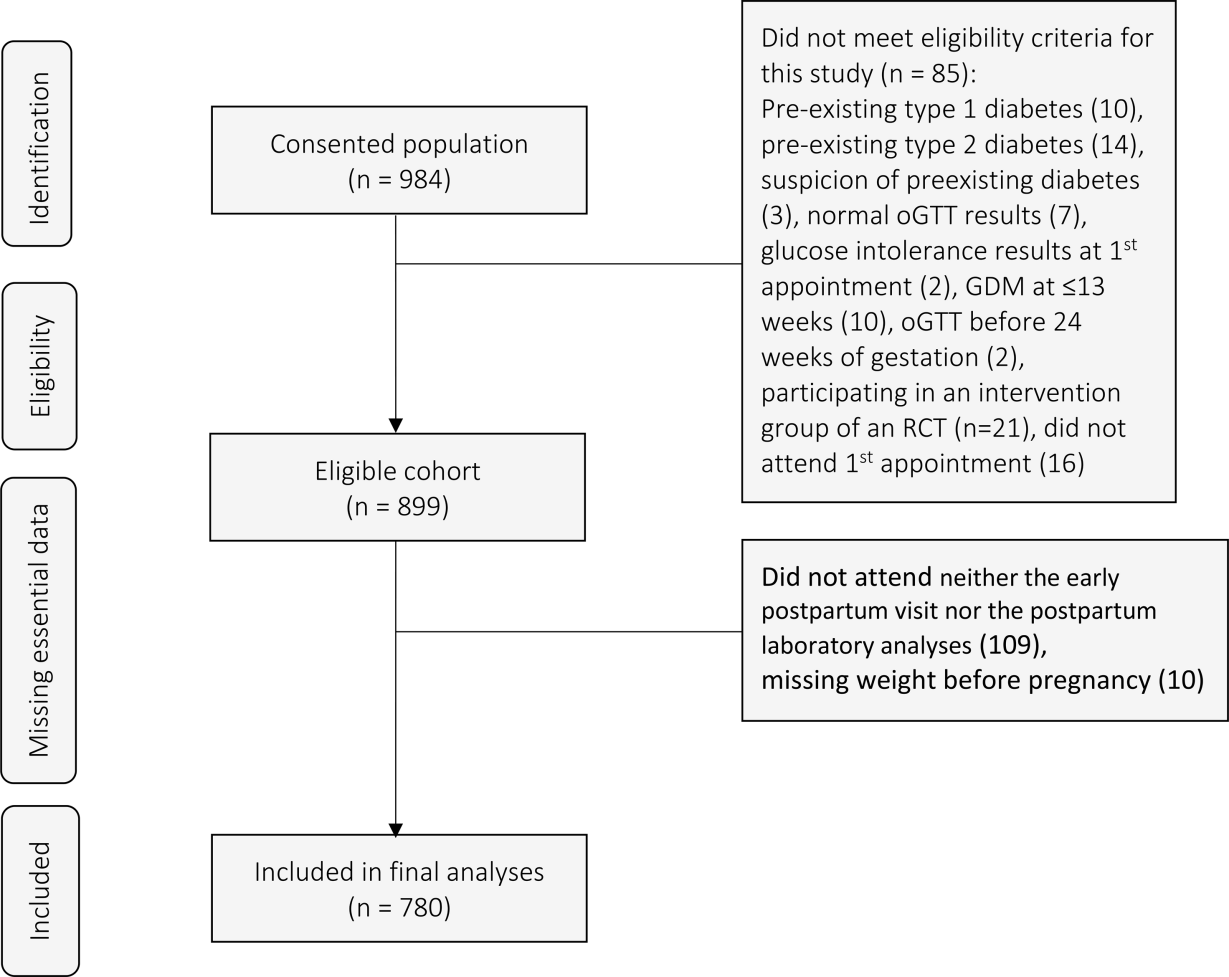
Flow chart of participating patients. oGTT, oral Glucose Tolerance Test; GDM, gestational diabetes mellitus; RCT, randomized controlled trial.

Out of the clinical population of 984 women who consented, we excluded 85 women who did not meet eligibility criteria of a clear definition of GDM, including also 16 women who did not attend their first scheduled appointment (**Figure 1**). We also excluded women who did not attend neither the early postpartum visit nor the postpartum laboratory analyses (n=109) and those with missing pre-pregnancy weight information (n=10). In the end, 780 pregnant women with GDM were included in the final analyses (**Figure 1**). Out of 780 women with GDM, 753 (97%) had available data for all four RF (27 missing data for ethnicity). Twenty-four percent (24%) (n=182) of women had no RF for GDM (**Table 1**). When nulliparous women were excluded (n=341), 18.3% of women in our cohort had no RF (RF-), 39.3% had one RF, 27.3% had two RF, 12.8% had three RF and 2.4% had more than three RF. The proportion of RF- women increased to 32% (n=254/780) when BMI ≥ 25 kg/m (2) counted only in combination with other adverse parameters (such as ethnicity, family history or GDM history) as a valid risk factor (6). When comparing RF- women and women with at least one RF (RF+), all descriptive characteristics except for maternal age and excessive GWG up to presentation at GDM clinic were found to be significantly different between the two groups (all p ≤ 0.01). Gestational age at delivery was similar between the two groups [38.2 (±2.5) weeks in RF- women vs 38.6 (±1.6) weeks in RF+ women, p=0.826]. There were no significant differences in the number of RF in women attending or not attending the postpartum visits (n=709 at 6-8 weeks and n=171 at 1 year post-partum, p 0.69 and 0.46 respectively).

**Table 2** shows the prevalence of maternal and neonatal outcomes according to the presence or absence of RF. The prevalence of severe maternal and neonatal outcomes was high in RF- women with 40% of them needing pharmacological treatment, 37% C-section delivery and 21% and 27% with glucose intolerance in early and late post-partum period respectively. RF+ women had higher glycaemic values at presentation, the end of pregnancy and in the early and late postpartum compared to RF- women and needed more frequently glucose-lowering medical treatment (all p ≤ 0.037 except for impaired glucose tolerance (IGT) in the early postpartum, p=0.05). Overall, 12% (n=46) were treated with metformin only, 5% (n=19) with a combination of metformin and insulin and 83% (n=317) with insulin alone. Although overall glucose intolerance was already 21% and 27% in the early and late postpartum in RF- women, this was increased by 2.1-2.9-fold in RF+ women (all p≤ 0.037, see above). In terms of obstetrical outcomes, RF- had higher rates of pre-eclampsia (4% vs 1% in RF+ women, p=0.031) but there were no differences in C-section delivery, PIH or in the composite outcome of maternal complications. When we adjusted for parity, gestational age at presentation and gestational age at delivery, all results remained unchanged except for pre-eclampsia, which lost its significance [OR 0.51 (95% CI 0.25-1.04), p=0.07)].

In terms of neonatal outcomes, RF+ women had almost twice the proportion of LGA (p=0.019), but less frequent preterm delivery (p=0.015). This difference was mostly driven by high-risk ethnicities (**Table 3**) without any differences in neonatal hypoglycaemia or the composite neonatal complications.

Regarding the impact of each of the four RF [GDM in previous pregnancy, FH T2DM, high-risk ethnicity and OW/OB before pregnancy (**Table 3**)] on short and long-term maternal and neonatal outcomes, OW/OB before pregnancy showed a significant impact on the majority of outcomes. This included the need for pharmacological treatment, PIH, HbA1c during pregnancy, C-section delivery, LGA, and overall glucose intolerance in the early and late postpartum. High-risk ethnicity was associated with reduced risk for preterm delivery, especially with but increased risk for overall glucose intolerance in the early and late postpartum. GDM in previous pregnancy showed an impact on HbA1c during pregnancy, on composite maternal complications, and on overall glucose intolerance in the early postpartum and FH T2DM on increased need for pharmacological treatment.

**Table 4** shows the cumulative impact of increasing the number of RF on each maternal outcome. The addition of each risk factor was associated with an increased risk for worsened adverse, particularly maternal metabolic outcomes except C-section delivery, PIH and composite maternal complications. The risk for overall glucose intolerance in the late postpartum increased by 1.7 with an additional risk factor resulting in a cumulative increased risk of 6.8 in the presence of all 4 RF compared to those with no RF(p=0.001). In contrast, the presence of more RF was associated with a reduced risk for pre-eclampsia (p=0.04). For neonatal outcomes, the cumulative impact of RF increased the risk for LGA, reduced the risk for preterm delivery (both p ≤ 0.025), and had no impact on the other outcomes.

When nulliparous women were excluded from the stepwise regression analysis, the cumulative impact of the number of RF on short and long-term maternal outcomes was similar except that HbA1c at the end of pregnancy and LGA did not remain significant (p=0.2 and p=0.6 respectively) (**Supplementary Table 1**).

When excessive GWG up to presentation at GDM clinic was added as an independent risk factor (**Supplementary Table 2**), the prevalence of RF- women decreased from 24% to 6% (n=43, p < 0.001). Maternal and neonatal outcomes were similar when excessive GWG up to presentation at GDM clinic was included except for loss in significance for the differences in pre-eclampsia and abnormal IGT in early post-partum (**Supplementary Table 2**). However, when we included GWG, the composite neonatal outcome became significantly different and was higher in RF- compared to RF+ women (p = 0.01).

## Discussion

This prospective cohort study explored the impact of RF on perinatal and postpartum outcomes in women with GDM in a clinical setting. We demonstrated that RF- women had a high prevalence of adverse maternal and neonatal outcomes despite a clinical follow-up. The presence of RF had a particular impact on overall glucose intolerance in the early and late postpartum. Pre-pregnancy OW/OB was a main predictor for both perinatal and postpartum outcomes. Finally, an accumulation of RF was associated with a gradual increase in adverse outcomes, particularly the need for pharmacological treatment, LGA and overall postpartum glucose intolerance, while pre-eclampsia and preterm delivery were reduced.

The prevalence of RF- women in our cohort is similar to those found in a recent multi-ethnic Belgian study (24% in our cohort vs 25.6% in Benhalima et al) (5). Even though all women in our cohort regardless of the number of RF received a regular follow-up and lifestyle advice, the prevalence of adverse maternal outcomes in RF- women was still high. The need for a pharmacological treatment was higher in our study than in other studies (40-54% in our study vs 23% in Benhalima et al. (30) and 27-30% in Alves et al. (14) studies respectively) which may be related to the elevated prevalence of high-risk ethnicities and family history of diabetes in the current cohort. Moreover, pregravid obesity has an impact on excessive fetal growth that can be attenuated by appropriate and early initiation of medical therapy (31–33).

Moreover, the prevalence of glucose intolerance in RF- women was 2-4 fold increased compared to the prevalence described in healthy cohorts of similar age (34, 35). Nevertheless, the incidence of most adverse maternal outcomes was higher in RF+ women compared to those RF-. This was not the case for C-section delivery, which might be dependent on the obstetrician and the diagnosis of GDM, and not just a protocol decision, nor for pre-eclampsia and preterm delivery. When adjusted for parity and gestational age at delivery and gestational age at presentation, preeclampsia was no longer significantly reduced in RF+ women. The reduced risk for preterm delivery in RF+ women might be explained by the lower risk found in non-Caucasian ethnicities. Indeed, preterm delivery was no longer reduced in RF+ women when adjusted for ethnicity (p=0.16, data not shown).

In a study conducted by Benhalima et al., the authors showed that as high as 33% of cases of GDM were missed when selective screening guidelines were applied (5). Recently, the ADA recommendations were modified and OW/OB was added as a risk factor in combination with other RF (36). In our cohort, we chose to analyse OW/OB as an independent risk factor. Most importantly, OW/OB is a modifiable GDM risk factor that had a considerable impact on most maternal outcomes and on LGA. When adapted to the new ADA definition (6), the prevalence of adverse maternal outcomes in the absence of RF would be even higher than what we have reported (**Table 2**). RF+ women had higher prevalence of overall glucose intolerance in early and late postpartum compared to their RF- counterparts. Our results are consistent with other studies that reported the general prevalence of glucose intolerance after GDM (14, 30) but higher than what was reported in an Irish study with a mean follow-up of 2.6 years (46% vs 18%) (37).

We found that previous GDM and particularly OW/OB were major RF associated with adverse outcomes. In our study, the odds of overall glucose intolerance in the early or late postpartum period were 1.7 and 2.4 times higher in OW/OB women. Although previous studies did not compare the respective importance of different RF, our data regarding the role of OW/OB as an independent risk factor for adverse maternal outcomes in women with GDM is in line with previous data (1, 14, 38). These previous studies reported that higher pre-pregnancy BMI was associated with higher risk of developing type 2 diabetes after pregnancy (1, 14, 38). Other RF such a previous GDM and high risk ethnicity have also been significantly linked to a higher risk of developing glucose intolerance and diabetes after GDM (14, 39–41). In our cohort, previous GDM and high-risk ethnicity were particularly associated with adverse outcomes in the postpartum period whereas FH T2DM was not as important in women already diagnosed with GDM. As OW/OB and excessive GWG up to presentation at GDM clinic represent the only modifiable established RF, they constitute an important target to change outcomes. As previous GDM is one of the most important RF for development of GDM, all women with previous GDM regardless of the presence of other RF should receive follow-up to detect and treat diabetes and also glucose intolerance (9). On the other side, our data also suggest that the cumulative presence of several RF is associated with a higher prevalence of adverse, mostly metabolic outcomes and thus the number of RF should inform the intensity of long-term follow-up in women with GDM.

The strengths of our study include our prospective design and the follow-up within usual clinical care. The multi-ethnic background of our population and the high rate of adherence to early postpartum testing (91%) increase the generalizability of our findings. Limitations of our study include the relatively low proportion of women (22%) followed until 1-year postpartum (as the 1-year follow-up started in 2015) and the absence of a control population. However, the glucose intolerance results at 6-8 weeks postpartum and 1 year postpartum are very similar even if outcomes were evaluated at the end of the follow-up. Other known RF for postpartum glucose intolerance that are not included in the recommendation of international societies were pre-pregnancy RF (maternal age, age of menarche, multiparity), glycaemic values of the oGTT, gestational weight gain and need for insulin treatment during pregnancy (5, 16–18) could be considered, but for reasons of simplicity they were not added in our analyses. We did not include maternal age (≥ 35 years) as a risk factor, because it is not part of the ADA recommendations but this could be a helpful tool for selective screening. However, the inclusion of women aged ≥ 35 years, (74 women) did not significantly change the results. Finally, our population was had a high prevalence of high-risk ethnicities and family history of diabetes. This, however, also reflects the multiethnicity of the population in Switzerland.

## Conclusion

We found that, among women with GDM, even those without diabetes-related RF had a high prevalence of adverse perinatal and postpartum outcomes. Most of these outcomes were more prevalent (%) in RF+ women and increased with increasing numbers of RF. Based on our results, postpartum follow-up should be proposed to all women with GDM regardless of the presence or absence of RF. OW/OB status was strongly associated with adverse perinatal and maternal complications, especially with adverse long-term metabolic outcomes. These women should be considered as a priority target during and after pregnancy as OW/OB, but also excessive GWG up to presentation at GDM clinic could be altered by lifestyle changes. High priority should be given to women with several RF to promote more intense and personalized patient-centred care.

## Data Availability Statement

The raw data supporting the conclusions of this article will be made available by the authors, without undue reservation.

## Ethics Statement

The studies involving human participants were reviewed and approved by The Human Research Ethics Committee of the Canton de Vaud (No. 326/15) approved the study protocol. The patients/participants provided their written informed consent to participate in this study.

## Author contributions

AC and JP wrote the first draft of the manuscript. AC, CK, DQ and JP had full access to the study data. All authors contributed to the interpretation of data, critically revised the manuscript and approved the final version for submission. JP is the guarantor of this work, and, as such, takes full responsibility for the integrity of the data used in the analysis.

## Funding

This study is a pilot of a project grant by the Swiss National Science Foundation (SNF 32003B_176119). The cohort database received an unrestricted educational grant from Novo Nordisk. JP’s research is also supported by the Leenaards Foundation and the Vontobel Foundation. The SNF and Novo Nordisk had no role regarding the content of the original data or analyses or in the drafting of this manuscript.

## Conflict of Interest

The authors declare that the research was conducted in the absence of any commercial or financial relationships that could be construed as a potential conflict of interest.

## Publisher’s Note

All claims expressed in this article are solely those of the authors and do not necessarily represent those of their affiliated organizations, or those of the publisher, the editors and the reviewers. Any product that may be evaluated in this article, or claim that may be made by its manufacturer, is not guaranteed or endorsed by the publisher.

## Supplementary Material

Supplementary Table 1 Multivariate regression analysis of cumulative impact of the number of risk factors on short and long-term maternal outcomes with exclusion of nulliparous women. All analysis were adjusted for parity and gestational age at presentation. For HbA1c at presentation and at the end of pregnancy, data were available for n==298 and n=168, respectively. Early post-partum was defined as 6-8 weeks post-partum and late post-partum as 1 year post-partum. Glucose intolerance defined as fasting glucose ≥5.6mmol/l or glucose T120 ≥7.8mmol/l (only for early post-partum) or HbA1c ≥5.7% (39 mmol/mol). Preterm delivery defined as < 37 weeks. LGA = large for gestational age. Neonatal hypoglycaemia defined as ≤ 2.5 mmol/l. ^+^ Overall glucose intolerance includes women with prediabetes and in addition 14 cases of diabetes in the early postpartum and 5 cases in the late post-partum. ^a^ Maternal complications include various pregnancy related complications such as placenta praevia, thrombopenia,… ^b^ Composite neonatal complications include Apgar score at 5 minutes < 7 and admission to intensive care unit (data available for n=615).

Supplementary Table 2 Impact of the absence or presence of any risk factors on short and long-term maternal outcomes including excessive GWG up to presentation at GDM visit as a risk factor. Data presented as n (%) or mean (±SD). Nulliparous patient were included in the analysis, as results were similar when excluded. For HbA1c at presentation and at the end of pregnancy, data were available for n==298 and n=168, respectively. Early post-partum was defined as 6-8 weeks post-partum and late post-partum as 1 year post-partum. Glucose intolerance defined as fasting glucose ≥5.6mmol/l or glucose T120 ≥7.8mmol/l (only for early post-partum) or HbA1c ≥5.7% (39 mmol/mol). Preterm delivery defined as < 37 weeks. LGA = large for gestational age. Neonatal hypoglycaemia defined as ≤ 2.5 mmol/l. ^+^ Overall glucose intolerance includes women with prediabetes and in addition 14 cases of diabetes in the early postpartum and 5 cases in the late post-partum. ^a^ Maternal complications include various pregnancy related complications such as placenta praevia, thrombopenia,…^b^ Composite neonatal complications include Apgar score at 5 minutes < 7 and admission to intensive care unit (data available for n=615).
