# Supplementary material for: Impact of Risk Factors on Short and Long-Term Maternal and Neonatal Outcomes in Women With Gestational Diabetes Mellitus: A Prospective Longitudinal Cohort Study
Source: Front Endocrinol (Lausanne). 2022 Jun 20;13:866446. doi: 10.3389/fendo.2022.866446 (PMC9251201; doi:10.3389/fendo.2022.866446)
Supplement: Supplementary Table 1 — Multivariate regression analysis of cumulative impact of the number of risk factors on short and long-term maternal outcomes with exclusion of nulliparous women. All analysis were adjusted for parity and gestational age at presentation. For HbA1c at presentation and at the end of pregnancy, data were available for n==298 and n=168, respectively. Early post-partum was defined as 6-8 weeks post-partum and late post-partum as 1 year post-partum. Glucose intolerance defined as fasting glucose ≥5.6mmol/l or glucose T120 ≥7.8mmol/l (only for early post-partum) or HbA1c ≥5.7% (39 mmol/mol). Preterm delivery defined as < 37 weeks. LGA = large for gestational age. Neonatal hypoglycaemia defined as ≤ 2.5 mmol/l. + Overall glucose intolerance includes women with prediabetes and in addition 14 cases of diabetes in the early postpartum and 5 cases in the late post-partum. a Maternal complications include various pregnancy related complications such as placenta praevia, thrombopenia,… b Composite neonatal complications include Apgar score at 5 minutes < 7 and admission to intensive care unit (data available for n=615). [file DataSheet_1.pdf]

Supplementary table 1. Multivariate regression analysis of cumulative impact of the number of risk factors on short and long-term maternal outcomes with exclusion of nulliparous women

	**OR#/β-coefficient (95%CI)**	**p-value**
**Maternal outcomes**		
HbA1c at presentation, %	0.20 (0.09 – 0.31)	< 0.001
HbA1c at the end of pregnancy, %+	0.17 (- 0.0 – 0.34)	0.218
Need for pharmacological treatment	1.60# (1.0 – 2.7)	0.05
C-section delivery	1.20# (0.79 – 2.07)	0.81
Pregnancy induced hypertension	0.45# (0.13 – 1.55)	0.208
Pre-eclampsia	0.27# (0.07 – 1.11)	0.071
Composite maternal complications [a]	n/a	n/a
Overall glucose intolerance in early post-partum+	1.92# (1.02 – 3.62)	0.017
Overall glucose intolerance in late post-partum+	2.31# (0.63 – 8.42)	0.016
**Neonatal outcomes**		
Preterm delivery	0.39# (0.18 – 0.87)	0.021
LGA	0.98# (0.54 – 1.79)	0.956
Neonatal hypoglycaemia	1.26# (0.49 – 3.20)	0.623
Composite neonatal complications [b]	0.69# (0.29 – 1.61)	0.319

Legend:
All analysis were adjusted for parity and gestational age at presentation. Odds ratio (OR) are marked with #.
For HbA1c at presentation and at the end of pregnancy, data were available for n==298 and n=168, respectively.
Early post-partum was defined as 6-8 weeks post-partum and late post-partum as 1 year post-partum. Glucose intolerance defined as fasting glucose ≥5.6mmol/l or glucose T120 ≥7.8mmol/l (only for early post-partum) or HbA1c ≥5.7% (39 mmol/mol). Preterm delivery defined as < 37 weeks. LGA = large for gestational age. Neonatal hypoglycaemia defined as ≤ 2.5 mmol/l.
+ Overall glucose intolerance includes women with prediabetes and in addition 14 cases of diabetes in the early postpartum and 5 cases in the late post-partum.
[a] Maternal complications include various pregnancy related complications such as placenta praevia, thrombopenia, ,…
[b] Composite neonatal complications include Apgar score at 5 minutes < 7 and admission to intensive care unit (data available for n=615).

Supplementary table 2 Impact of the absence or presence of any risk factors on short and long-term maternal outcomes including excessive GWG up to presentation at GDM visit as a risk factor

	**No risk factor (n=43)**	**At least one risk factor (n=737)**	**OR#/β-coefficient (95% confidence interval)**	**p-value**
**Maternal outcomes**				
HbA1c at presentation, %	5.2 (0.3)	5.5 (0.4)	0.25 (0.12 – 0.39)	< 0.001
HbA1c at presentation, mmol/mol	33.5 (3.7)	36.3 (4.6)	2.78 (1.32 – 4.25)	< 0.001
HbA1c at the end of pregnancy, %+	5.3 (0.3)	5.5 (0.4)	0.20 (-0.02 – 0.42)	0.071
HbA1c at the end of pregnancy , mmol/mol	34.8 (3.6)	37.1 (4.3)	2.22 (-0.19 – 4.63)	0.071
Need for pharmacological treatment	11 (26%)	384 (52%)	3.16# (1.6 – 6.37)	< 0.001
C-section delivery	12 (33%)	279 (41%)	1.37# (0.67 – 2.79)	0.377
Pregnancy induced hypertension	1 (2%)	23 (3%)	1.35# (0.18 – 10.26)	0.770
Pre-eclampsia	1 (2%)	13 (2%)	0.75# (0.09 – 5.90)	0.788
Composite maternal complications [a]	1 (2%)	22 (3%)	1.29# (0.17 – 9.82)	0.804
**Overall glucose intolerance in the early postpartum+**	4 (13%)	221 (33%)	3.50# (1.21 – 10.10)	0.008
Abnormal fasting glucose at 6-8 weeks postpartum<br>Pre-diabetes (IFG)<br>Diabetes	1 (3%)<br>1 (3%)<br>0	98 (14%)<br>90 (13%)<br>8 (1%)	5.97# (0.81 – 44 .04)	0.019
Abnormal 2h glucose at 6-8 weeks postpartum<br>Pre-diabetes (IGT)<br>Diabetes	4 (12%)<br>3 (9%)<br>1 (3%)	54 (8%)<br>46 (7%)<br>8 (1%)	0.66# (0.22 – 1.94)	0.471
Abnormal HbA1c at 6-8 weeks postpartum<br>Pre-diabetes<br>Diabetes	2 (6%)<br>2 (6%)<br>0	152 (23%)<br>148 (22%)<br>4 (1%)	4.67# (1.11 – 19.74)	0.008
**Overall glucose intolerance in the late postpartum+**	1 (12%)	78 (48%)	6.42# (0.77 – 53.39)	0.036
Abnormal fasting glucose at 1 year postpartum<br>Pre-diabetes (IFG)<br>Diabetes	1 (12%)<br>1 (12%)<br>0	70 (42%)<br>66 (40%)<br>4 (2%)	5.27# (0.63 . 43.81)	0.066
Abnormal HbA1c at 1 year postpartum<br>Pre-diabetes<br>Diabetes	0<br>0<br>0	28 (17%)<br>24 (15%)<br>4 (2%)	n/a	n/a
**Neonatal outcomes**				
Preterm delivery	8 (21%)	63 (9%)	0.38# (0.17 – 0.87)	0.022
LGA	2 (5%)	112 (16%)	3.58# (0.85 – 15.06)	0.082
Neonatal hypoglycaemia	1 (2%)	63 (8%)	3.92# (0.53 – 29.00)	0.180
Composite neonatal complications [b]	9 (29%)	78 (12%)	0.34# (0.15 – 0.77)	0.01

Legend:
Data presented as n (%) or mean (±SD). Odds ratio (OR) are marked with #.
Nulliparous patient were included in the analysis, as results were similar when excluded.
For HbA1c at presentation and at the end of pregnancy, data were available for n==298 and n=168, respectively. Early post-partum was defined as 6-8 weeks post-partum and late post-partum as 1 year post-partum. Glucose intolerance defined as fasting glucose ≥5.6mmol/l or glucose T120 ≥7.8mmol/l (only for early post-partum) or HbA1c ≥5.7% (39 mmol/mol). Preterm delivery defined as < 37 weeks. LGA = large for gestational age. Neonatal hypoglycaemia defined as ≤ 2.5 mmol/l.

[+] Overall glucose intolerance includes women with prediabetes and in addition 14 cases of diabetes in the early postpartum and 5 cases in the late post-partum.

[a] Maternal complications include various pregnancy related complications such as placenta praevia, thrombopenia, ,…

[b] Composite neonatal complications include Apgar score at 5 minutes < 7 and admission to intensive care unit (data available for n=615).
